# Hepatic Glucose Uptake During Euglycemic Hyperinsulinemia Associates With Glycemia During Oral Glucose Tolerance Test

**DOI:** 10.1210/jendso/bvaf054

**Published:** 2025-04-02

**Authors:** Miikka-Juhani Honka, Eleni Rebelos, Laura Pekkarinen, Nelli Tuomola, Aino Latva-Rasku, Leena Koukkari, Heidi Immonen, Andrea Mari, Kari K Kalliokoski, Jarna C Hannukainen, Pirjo Nuutila

**Affiliations:** Turku PET Centre, University of Turku, Turku FI-20520, Finland; Turku PET Centre, Turku University Hospital, Turku FI-20520, Finland; Division of Information Science, Nara Institute of Science and Technology, Ikoma, Nara 630-0192, Japan; Turku PET Centre, University of Turku, Turku FI-20520, Finland; First Department of Propaedeutic Internal Medicine, Diabetes Center, Medical School, National and Kapodistrian University of Athens, Laiko General Hospital, Athens 11527, Greece; Department of Clinical and Experimental Medicine, University of Pisa, Pisa 56126, Italy; Turku PET Centre, University of Turku, Turku FI-20520, Finland; Department of Endocrinology, Turku University Hospital, Turku FI-20520, Finland; Turku PET Centre, University of Turku, Turku FI-20520, Finland; Department of Endocrinology, Turku University Hospital, Turku FI-20520, Finland; Turku PET Centre, University of Turku, Turku FI-20520, Finland; Department of Endocrinology, Turku University Hospital, Turku FI-20520, Finland; Turku PET Centre, University of Turku, Turku FI-20520, Finland; Turku PET Centre, University of Turku, Turku FI-20520, Finland; Department of Endocrinology, Turku University Hospital, Turku FI-20520, Finland; Institute of Neuroscience, National Research Council, Padova 35127, Italy; Turku PET Centre, University of Turku, Turku FI-20520, Finland; Turku PET Centre, University of Turku, Turku FI-20520, Finland; Turku PET Centre, Turku University Hospital, Turku FI-20520, Finland; Turku PET Centre, University of Turku, Turku FI-20520, Finland; Department of Endocrinology, Turku University Hospital, Turku FI-20520, Finland

**Keywords:** liver, glucose uptake, positron emission tomography, euglycemic hyperinsulinemic clamp, insulin resistance, oral glucose tolerance test, [^18^F]fluorodeoxyglucose, glucokinase

## Abstract

**Context:**

Postprandial hepatic glycogen synthesis and glycolysis are reduced in hepatic insulin resistance. However, the physiologic interpretation of the reduction in hepatic glucose uptake (GU) during the gold-standard measurement of insulin sensitivity, hyperinsulinemic euglycemic clamp, in insulin resistance is unclear. This is because the peripheral route of glucose and insulin delivery during a clamp study differs greatly from the physiological route.

**Objective:**

We hypothesized that hepatic GU during hyperinsulinemic euglycemic clamp would predict glycemia during oral glucose tolerance test (OGTT).

**Design:**

We analyzed cross-sectional data of 120 individuals (70 men and 50 women) who did not have diabetes from the CMgene study cohort. Hepatic GU was measured with [^18^F]fluorodeoxyglucose ([^18^F]FDG) and positron emission tomography.

**Results:**

In a multiple regression analysis, hepatic GU, endogenous glucose production, insulin secretion capacity, and serum triglycerides predicted OGTT glucose area under the curve (*P* for all <.05), whereas skeletal muscle GU, the antilipolytic insulin index, and insulin clearance were not statistically significant predictors (*P* > .05).

**Conclusions:**

Hepatic GU measured during hyperinsulinemic euglycemic clamp is an independent predictor of OGTT glucose area under the curves even when accounting for well-known other factors affecting glycemic control. This finding supports the idea that insulin-mediated hepatic GU, and more broadly, first-pass glucose extraction, have a meaningful contribution to glycemic control. Thus, this measurement provides useful information about hepatic insulin sensitivity in the more physiologic conditions of the OGTT which may be useful when studying the pathophysiology of impaired glucose tolerance and when evaluating potential treatments for impaired glycemic control.

Dysregulation in postprandial glycemic control is traditionally attributed to reflect impairments in insulin-mediated suppression of endogenous glucose production, stimulation of glucose uptake (GU) into skeletal muscles, and insulin secretion, whereas insulin resistance of hepatic GU has been thought to have little importance [1]. The interpretation of minor role of hepatic GU is based on studies where splanchnic glucose balance was evaluated by hepatic vein catheterization and stable glucose tracers during the gold-standard measurement of insulin sensitivity, hyperinsulinemic euglycemic clamp (HEC), that showed insulin to have little stimulatory effect on splanchnic GU [1]. We and others showed later by employing 2-deoxy-2-[^18^F]fluoro-D-glucose ([^18^F]FDG), positron emission tomography (PET), and HEC that hepatic GU actually is insulin sensitive in humans and is reduced in insulin resistant conditions [2-5]. The strength of using PET is that it allows the measurement of hepatic GU directly whereas the arteriovenous method used in the earlier studies included also other splanchnic organs because of inaccessibility of the portal vein in humans. In these PET studies, hepatic [^18^F]FDG net influx rates correlated to fasting glucose and hemoglobin A1c (HbA1c) measurements suggesting that insulin-stimulated hepatic GU has a role in glycemic control [2-4]. However, because these studies only investigated bivariate correlations (ie, not accounting for confounders), it is possible that association with glycemic control in these studies was driven by other factors that correlate both with hepatic GU and glycemic measures, such as skeletal muscle GU. In addition, fasting glucose and HbA1c are not ideal markers for postprandial glycemia [6], where hepatic GU contributes more on glucose clearance than in fasting [1].

Quantification of tissue GU using [^18^F]FDG is based on its property of being trapped in the cells as [^18^F]FDG-6-phosphate after phosphorylation by a hexokinase [7, 8]. As liver glucose transporters have high capacity, the hepatic GU measurement with [^18^F]FDG mostly reflects glucokinase activity, that has been shown to be the rate-limiting step in hepatic glycogen synthesis, a process which is impaired in hepatic insulin resistance [9, 10]. In addition, circulating lactate concentrations after glucose ingestion, which reflect hepatic glycolysis, are less increased in insulin resistance suggesting impaired glucokinase activity [11, 12].

To shed light on the physiologic relevance of the insulin-stimulated hepatic GU measurement, we evaluated the contribution of this measure on the glucose curves during an oral glucose tolerance test (OGTT) using linear regression. We hypothesized that insulin-stimulated hepatic GU would be an independent predictor of the glucose curves during an OGTT even when accounting for factors known to contribute to glycemic control: skeletal muscle GU, suppression of endogenous glucose production (EGP), the antilipolytic effect of insulin, insulin secretion capacity, and insulin clearance [13-17].

## Methods

### Participants

This cross-sectional study included 120 subjects (Table 1) who did not have diabetes from the CMgene study cohort consisting of previous PET studies conducted at Turku PET Centre (Turku, Finland) (ClinicalTrials.gov Identifier: NCT03310502). The inclusion of participants was based on availability of OGTT with sampling at multiple timepoints and measurement of hepatic GU and factors known to affect glycemic control (skeletal muscle GU, suppression of EGP, the antilipolytic effect of insulin, insulin secretion capacity, and insulin clearance; all of these measurements were required for the inclusion). The original PET/OGTT studies ([2, 19, 20], NCT05080205, NCT06739473, and healthy controls in NCT05101538) were performed during the years 2009 to 2024. The current study included only measurements from the baseline of these PET/OGTT studies (before any intervention) to avoid confounding effects from the treatments. The median interval between the OGTT and PET study was 33 days (interquartile range 15, 88 days). The participants (70 men and 50 women) were young and middle-aged adults (median age 37.5, range 20-60 years), and presented a wide range of body mass index (BMI; median 25, range 19-48 kg/m^2^), insulin sensitivity (median M-value 32.9, range 5.7-86.1 µmol/kg body weight/min), and glycemic control (median fasting glucose 5.4 mmol/L, range 3.0-6.85 mmol/L; median OGTT 2 hours glucose 5.9, range 2.9-10.3 mmol/L). Women were older than men (median age 39 years, range 20-60 vs 34, 20-55) but had a comparable BMI 25, 19-45 vs 25, 19-48 kg/m^2^. None of the included participants had metformin or any other diabetes medication. One participant had anxiolytic and 3 had antidepressant medication, 8 participants had antihypertensive medication, 2 had statin medication, 11 had estrogen, 13 had progesterone medication, and 1 had testosterone medication. All subjects gave a written informed consent for participating in the study. The study protocol was approved by the Ethics Committee of the wellbeing services county of Southwest Finland.

**Table 1.** Descriptive characteristics of the study participants

	Low OGTT glucose_AUC_	High OGTT glucose_AUC_	*P* value
n (NGT/iIFG/iIGT/cIFG-IGT)	45/15/0/0	22/22/6/10	
N (men/women)	60 (40/20)	60 (30/30)	.010
Age (y)	35 (24; 45)	41 (28; 50)	.054
BMI (kg/m^2^)	25 (22; 28)	26 (24; 33)	.024
Waist-to-hip ratio	0.88 (0.81; 0.94)	0.92 (0.82; 0.98)	.144
Fasting plasma glucose (mmol/L)	5.2 (5.0; 5.6)	5.6 (5.3; 6.0)	8.8E-5
Fasting plasma insulin (pmol/L)	33 (24; 47)	54 (30; 77)	1.5E-4
OGTT 120 min plasma glucose (mmol/L)	5.2 (4.2; 5.7)	7.2 (6.0; 7.9)	4.6E-13
OGTT 120 min plasma insulin (pmol/L)	132 (90; 192)	336 (156; 504)	3.8E-11
OGTT glucose_AUC_ (mmol min/L)	711 (653; 784)	963 (891; 1082)	
HbA1c (%)	5.3 (4.9; 5.6)	5.5 (5.4; 5.8)	2.1E-4
HOMA-IR	1.3 (0.9; 1.7)	2.1 (1.3; 3.1)	3.1E-5
Endogenous glucose production (µmol/body weight kg/min)	−0.5 (−7.4; 3.4)	4.1 (0.2; 8.6)	1.7E-5
Insulin_AUC0-30_/glucose_AUC0-30_ (pmol/mmol)	21.3 (15.0; 38.0)	28.2 (18.1; 38.4)	.262
Skeletal muscle GU (μmol/tissue kg/min)	53.1 (36.6; 68.3)	30.3 (21.1; 58.0)	3.3E-4
Hepatic GU (µmol/tissue L/min)	21.2 (16.6; 30.3)	20.4 (14.5; 26.1)	.162
Antilipolytic insulin index (pmol/L*mmol/L)	17.6 (9.7; 26.4)	25.2 (15.1; 38.0)	.005
Insulin clearance, clamp (L/m^2^/min)	0.58 (0.49; 0.68)	0.55 (0.47; 0.62)	.147
M-value (µmol/body weight kg/min)	42.7 (30.7; 57.3)	26.5 (17.1; 37.0)	1.0E-6
Results from OGTT modeling			
Total insulin secretion (nmol/m^2^)	35.2 (28.6; 42.6)	44.0 (37.3; 58.0)	5.0E-6
Glucose sensitivity (pmol/min/m^2 ^L/mmol)	117 (83; 178)	88 (69; 117)	.013
Rate sensitivity (pmol/m^2^ L/mmol)	636 (431; 1090)	787 (547; 1146)	.372
Potentiation factor ratio	2.08 (1.36; 2.59)	1.39 (1.11; 1.80)	5.7E-4
Mean insulin clearance (L/m^2^/min)	1.42 (1.19; 1.68)	1.18 (0.97; 1.40)	8.5E-4

To illustrate the differences between individuals with low or high oral glucose tolerance test (OGTT) glycemia, the study population is split to low and high OGTT glucose area under the curve (AUC) groups according to the median value (below/equal and above 819 mmol min/L) in this table.

Glucose tolerance status was determined by using the classification by the American Diabetes Association [18]. Of the OGTT-derived parameters glucose sensitivity describes glucose-sensitive insulin secretion, rate sensitivity describes secretion response to rate of change in glucose concentration, and potentiation factor ratio describes insulin secretion response to potentiating factors, including incretins. Data presented as median (1st quartile; 3rd quartile).

Abbreviations: cIFG-IGT, combined impaired fasting glucose and impaired glucose tolerance; GU, glucose uptake; HbA1c, hemoglobin A1c; HOMA-IR, homeostatic model assessment for insulin resistance; iIFG, isolated impaired fasting glucose; iIGT, isolated impaired glucose tolerance; NGT, normal glucose tolerance.

### PET and OGTT Measurements

Details of the PET study design and methods are described in the Supplementary methods [21]. In brief, hepatic and femoral skeletal muscle GU were measured during a 40 mU/m^2^/min HEC using PET in combination with [^18^F]FDG [22] and the antilipolytic effect of insulin was measured by the product of insulin and free fatty acid concentrations during the clamp study [23]. In addition, we included fasting triglycerides as a measure of fatty acid availability to the model. EGP was measured by subtracting glucose infusion rate during the clamp study from the glucose rate of disappearance measured using [^18^F]FDG plasma clearance [24]. OGTT was performed using a 75-g glucose dose. Plasma glucose, insulin, and C-peptide concentrations were measured at 0, 30, 60, 90, and 120 minutes and the area under the curve (AUC) for OGTT glucose was determined. The insulin_AUC0-30_/glucose_AUC0-30_ ratio from OGTT was used as a measure of insulin secretion [25, 26]. Insulin clearance was measured by insulin infusion rate/steady-state insulin concentration ratio [3, 27], where the contribution of peripheral (kidneys, skeletal muscle, and adipose tissue) insulin clearance is proportionally pronounced because peripherally infused insulin bypasses the hepatic first-pass insulin extraction [28]. In addition, we performed mathematical modeling to measure β-cell glucose sensitivity, rate sensitivity, potentiation, and insulin clearance from OGTT data (described in more detail in the supplementary methods) [21, 29, 30]. Homeostatic Model Assessment for Insulin Resistance was calculated as HOMA-IR = fasting plasma glucose (mmol/L) × fasting plasma insulin (mU/L)/22.5 as previously described [31].

### Statistical Analysis

Factors contributing to OGTT glucose AUCs were evaluated using multiple regression. The included variables were ascertained to have variance inflation factor less than 5 and tolerance above 0.25 to avoid issues with multicollinearity (these thresholds were not used when testing the effects of interaction with sex because interactions are correlated with the original variables). The number of 10 independent variables in a regression analysis was used as the basis when evaluating the required sample size (liver GU, skeletal muscle GU, suppression of EGP, the antilipolytic effect of insulin, serum triglycerides, insulin secretion capacity, and insulin clearance plus age, sex, and BMI to account for confounding). The achieved sample size 120 was considered sufficient for multiple regression based on a previous simulation study where a sample size of ≥10 subjects per independent variable yielded reliable estimates of *R*^2^ and regression coefficients [32]. Logarithmic or square root transformation was used for variables to linearize the relationship between the dependent and independent variables in the model where needed (the used transformations are mentioned in the regression result tables). Three cases in the data had high median absolute deviation > |±3| for EGP [33]; these were adjusted to median absolute deviation = |±3| to achieve normal distribution [34]. T-test or Mann-Whitney *U* test was used for comparing the groups of low and high OGTT glucose AUCs (based on splitting the data by the median of glucose_AUC_) (Table 1) or for the comparison of sexes (Supplementary Table S1 [21]) for descriptive statistics. Proportions of variance explained are reported using adjusted *R*^2^s. The data were analyzed using IBM SPSS Statistics for Windows, Version 27.0. (IBM Corp, Armonk, NY). *P* values <.05 were considered statistically significant.

## Results

### Descriptive Characteristics

The participants with low glucose_AUC_ (below median) had lower BMI, better suppression of EGP and lipolysis, and higher skeletal muscle GU and whole body insulin sensitivity than participants with high glucose_AUC_ (above median) (Table 1). In addition, age tended to be lower among the low glucose_AUC group_ (*P* = .054). Despite the differences in BMI and age, there was a good overlap in these variables between the 2 groups (range of BMI 19-45 vs 19-48 kg/m^2^ and range of age 20-60 vs 20-57 years in low vs high glucose_AUC_ group, respectively). Insulin secretion capacity as measured by insulin_AUC0-30_/glucose_AUC0-30_ ratio showed no difference between these groups. On the other hand, results from mathematical modeling of β-cell function showed lower glucose sensitivity and higher total insulin secretion among the participants with high OGTT glucose_AUC_. Thus, the higher insulin secretion was proportionally matched to the increased glucose concentrations in individuals with high glucose_AUC_. In addition, potentiation factor ratio was lower among participants with high OGTT glucose AUCs consistently with the known role of potentiation in regulating late glucose concentration [35, 36]. In addition, insulin clearance as measured during euglycemic hyperinsulinemia or OGTT did not differ between the groups.

### Primary Regression Finding

We ran multiple regression to predict OGTT glucose AUC using hepatic GU, skeletal muscle GU, suppression of EGP, antilipolytic effect of insulin, insulin secretion capacity, and insulin clearance as independent variables in the model. The model explained 30% in variation in OGTT glucose AUC (*P* = 3.0E-8) and EGP, insulin secretion capacity, hepatic GU, and fasting triglycerides were statistically significant predictors in the model (Table 2).

**Table 2.** The primary regression model: the model predicts oral glucose tolerance test glucose area under the curve from 0 to 120 min (AUC; mmol/L*min)*^a^*

	Unstandardized coefficients	95% CI		Standardized coefficients	*P* value
	B	Lower	Upper	β	
(Constant)	36.083	31.440	40.851		<.001
Endogenous glucose production (µmol/body weight kg/min)	0.123	0.065	0.188	0.320	<.001
Insulin_AUC0-30_/glucose_AUC0-30_ (pmol/mmol)*^b^*	−3.193	−5.819	−0.978	−0.300	.010
Hepatic GU (µmol tissue L^−1^ minute^−1^)*^a^*	−0.548	−0.937	−0.065	−0.193	.014
Skeletal muscle GU (μmol tissue kg^−1^ minute^−1^)*^a^*	−0.249	−0.0.593	0.035	−0.160	.119
Antilipolytic insulin index (pmol/L*mmol/L)*^b^*	1.034	−0.890	2.926	0.102	.281
Insulin clearance (L m^−2^ minutes^−1^)*^b^*	−0.770	−6.128	4.549	−0.024	.771
Fasting triglycerides (mmol/L)*^b^*	4.295	1.629	6.531	0.271	.001

The standardized regression coefficients in the model serve as intuitive indices of effect size: higher absolute value for a coefficient indicates larger effect on glucose AUCs.

Abbreviation: GU, glucose uptake.

^a^Square root transformed variable.

^b^log_10_-transformed variable.

Insulin sensitivity of EGP suppression can be also expressed by the EGP*insulin product [37]; however, replacing EGP by EGP*steady state insulin in the regression analysis yielded practically the same model as in Table 2 with similarly sized standardized regression coefficients for each measure (data not shown).

### Confirmatory Regression Analyses

To confirm that the results in Table 2 represented actual effects of the individual variables and were not modified because of some confounding factor, we ran additional multiple regression analyses with variables that could have modified the results. First, BMI correlated with OGTT glucose AUCs, EGP, insulin_AUC0-30_/glucose_AUC0-30_, liver and muscle GU, antilipolytic insulin index, insulin clearance, and fasting triglycerides (*P* < .01 for all) and age correlated with OGTT glucose AUCs, EGP, antilipolytic insulin index, and fasting triglycerides (*P* < .05) and there was a significant difference between the sexes in OGTT 2-hour glucose, insulin clearance, and M-value (Supplementary Table S1 [21]). Thus, although not directly involved, it is possible that age, sex, or BMI could modify the relationships described in Table 2. Including age, sex, and BMI lead to a small increase in the variance explained by the model (*R*^2^ = 34%, *P* = 1.1E-8 for the model) and sex explained variation beyond the insulin sensitivity and secretion measures that affect the glucose levels directly (Table 3). Because sex was a statistically significant predictor when added to the primary model, we performed a separate regression analysis inspecting the possible modifying effect of sex on the association with OGTT glucose AUCs. In this analysis, interaction terms were created by multiplying the original variables from Table 2 by the dichotomous variable sex (male = 0; female = 1). Including these terms improved performance of the model (*R*^2^ = 40%, *P* = 4.2E-9 for the model), and we found that sex had a significant interaction effect with insulin clearance during euglycemic hyperinsulinemia (*P* = .043; lower coefficient in women) and a trend for an interaction effect with antilipolytic effect of insulin (*P* = .072; lower coefficient in women) and serum triglycerides (*P* = .092; lower coefficient in women) (Supplementary Table S2 [21]). These interaction terms and sex are included in the confirmatory analyses below to account for their effect (predictors in the reference Confirmatory model 1: EGP, Insulin_AUC0-30_/Glucose_AUC0-30,_ hepatic GU, skeletal muscle GU, antilipolytic insulin index, insulin clearance, fasting triglycerides, sex*antilipolytic insulin index, sex*insulin clearance, sex*fasting triglycerides, sex; *R*^2^ = 39%, *P* = 5.4E-10, Table 4). Furthermore, 2 participants of the study had statin, 8 had antihypertensive, 1 had anxiolytic, 3 had antidepressive, 11 estrogen, 13 progesterone, or 1 testosterone medication (30 participants combined). The number of users for statin, anxiolytic, antidepressive, or testosterone medications were too small to use these medications as covariates in a regression analysis. Nevertheless, excluding these participants had minimal effect on the regression coefficients compared to the Confirmatory model 1, suggesting that the medications did not cause meaningful confounding on the results (data not shown). Similarly, including estrogen, progesterone, and antihypertensive medication as covariates to the Confirmatory model 1 had little effect on the standardized regression coefficients, but having antihypertensive medication was associated with an increase in OGTT glucose AUC (*P* = .038 for the medication's coefficient). Moreover, previous evidence suggests that blood pressure may be causally related to glycemic control [38]. However, adding either systolic or diastolic blood pressure to the Confirmatory model 1 had little effect on the model coefficients (data not shown). There is also evidence from a Mendelian randomization study that serum lipids are causally related to glycemic control [39]. However, neither low-density protein cholesterol nor high-density lipoprotein predicted OGTT glucose AUC when added to Confirmatory model 1 (data not shown).

**Table 3.** Regression model predicting oral glucose tolerance test glucose (OGTT) area under the curve from 0 to 120 min (AUC; mmol/L*min)*^a^*

	Unstandardized coefficients	95% CI		Standardized coefficients	*P* value
	B	Lower	Upper	β	
(Constant)	35 784	26.967	44.163		<.001
Endogenous glucose production (µmol/body weight kg/min)	0.121	0.061	0.180	0.313	<.001
Insulin_AUC0-30_/glucose_AUC0-30_ (pmol/mmol)*^b^*	−3.304	−5.759	−1.159	−0.310	.005
Hepatic GU (µmol tissue l^−1^ minute^−1^)*^a^*	−0.565	−0.969	−0.072	−0.199	.014
Skeletal muscle GU (µmol tissue kg^−1^ minute^−1^)*^a^*	−0.286	−0.684	0.026	−0.184	.104
Antilipolytic insulin index (pmol/L*mmol/L)*^b^*	0.450	−1.656	2.533	0.044	.670
Insulin clearance (L m^−2^ minutes^−1^)*^b^*	−3.534	−9.003	2.274	−0.110	.208
Fasting triglycerides (mmol/L)*^b^*	4.084	1.368	6.396	0.258	.003
Sex (M: 0; F: 1)	1.207	0.131	2.170	0.197	.022
Age (y)	0.033	−0.014	0.082	0.129	.171
BMI (kg/m^2^)*^b^*	−0.630	−6.204	5.291	−0.020	.829

This analysis was performed to study the potential confounding effect of sex, age, and BMI on the prediction of OGTT glucose AUCs. The standardized regression coefficients in the model serve as intuitive indices of effect size: higher absolute value for a coefficient indicates larger effect on glucose AUCs.

Abbreviations: BMI, body mass index; F, female; GU, glucose uptake; M, male.

^a^Square root transformed variable.

^b^log_10_-transformed variable.

**Table 4.** Confirmatory model 1: regression model predicting oral glucose tolerance test glucose area under the curve from 0 to 120 min (AUC; mmol/L*min)*^a^*

	Unstandardized coefficients	95% CI		Standardized coefficients	*P* value
	B	Lower	Upper	β	
(Constant)	34.334	29.255	39.137		<.001
Endogenous glucose production (µmol/body weight kg/minute)	0.134	0.071	0.197	0.348	<.001
Insulin_AUC0-30_/glucose_AUC0-30_ (pmol/mmol)	−3.418	−5.501	−1.326	−0.321	<.001
Hepatic GU (µmol tissue L^−1^ minute^−1^)*^a^*	−0.502	−0.878	−0.110	−0.177	.016
Skeletal muscle GU (μmol tissue kg^−1^ minute^−1^)	−0.236	−0.548	0.029	−0.152	.099
Antilipolytic insulin index (pmol/L*mmol/L)*^a^*	2.469	0.207	4.607	0.243	.027
Insulin clearance (L m^−2^ minutes^−1^)	1.345	−6.468	8.782	0.042	.745
Fasting triglycerides (mmol/L)*^b^*	5.898	2.729	8.344	0.372	.001
Sex*antilipolytic insulin index (pmol/L*mmol/L)*^b^*	−3.916	−6.849	−0.580	−0.861	.011
Sex*insulin clearance (L m^−2^ minutes^−1^)*^b^*	−10.739	−21.053	−0.319	−0.438	.042
Sex*fasting triglycerides (mmol/L)*^b^*	−4.264	−8.549	0.744	−0.169	.071
Sex (M: 0; F: 1)	3.648	−0.605	7.760	0.595	.070

This analysis is a reference for the confirmatory models predicting OGTT glucose AUCs. The standardized regression coefficients in the model serve as intuitive indices of effect size: higher absolute value for a coefficient indicates larger effect on glucose AUCs.

Abbreviations: F, female; GU, glucose uptake; M, male; OGTT, oral glucose tolerance test; R_d_, glucose rate of disappearance.

^a^Square root transformed variable.

^b^log_10_-transformed variable.

Even though skeletal muscle is the most important target organ for glucose disposal during a hyperinsulinemic clamp study, the contribution of other organs, such as adipose tissue or the brain can be considerable in an insulin resistant state [4, 40, 41]. In line with this, replacing skeletal muscle GU with the *M*-value or glucose rate of disappearance (*R*_d_) in Confirmatory model 1 resulted in similarly performing models (41%, *P* = 1.7E-10, Table 5 and 40%, *P* = 1.9E-10, Supplementary Table S3 [21], respectively) where both *M*-value and *R*_d_ were significant predictors.

**Table 5.** Regression model predicting oral glucose tolerance test glucose (OGTT) area under the curve from 0 to 120 min (AUC; mmol/L*min)*^a^*

	Unstandardized coefficients	95% CI		Standardized coefficients	*P* value
	B	Lower	Upper	β	
(Constant)	38.060	31.557	44.153		<.001
Endogenous glucose production (µmol/body weight kg/minute)	0.100	0.027	0.172	0.258	.004
Insulin_AUC0-30_/glucose_AUC0-30_ (pmol/mmol)	−3.705	−5.785	−1.535	−0.348	.001
Hepatic GU (µmol tissue L^−1^ min^−1^)*^a^*	−0.430	−0.811	−0.030	−0.152	.029
M-value (µmol body weight kg^−1^ minute^−1^)*^a^*	−0.636	−1.199	−0.072	−0.323	.037
Antilipolytic insulin index (pmol/L*mmol/L)*^a^*	1.134	−1.350	3.237	0.111	.326
Insulin clearance (L m^−2^ minutes^−1^)	−0.489	−8.824	7.569	−0.015	.892
Fasting triglycerides (mmol/L)*^b^*	5.790	2.967	8.473	0.365	<.001
Sex*antilipolytic insulin index (pmol/L*mmol/L)*^b^*	−3.413	−6.283	−0.100	−0.750	.025
Sex*insulin clearance (L m^−2^ minutes^−1^)*^b^*	−9.796	−19.711	1.467	−0.400	.072
Sex*fasting triglycerides (mmol/L)*^b^*	−4.476	−9.062	0.110	−0.178	.059
Sex (M: 0; F: 1)	2.659	−1.549	6.359	0.434	.187

This analysis was performed to study if the inclusion of M-value would improve prediction of OGTT glucose AUC. The standardized regression coefficients in the model serve as intuitive indices of effect size: higher absolute value for a coefficient indicates larger effect on glucose AUCs.

Abbreviations: F, female; GU, glucose uptake; M, male.

^a^Square root transformed variable.

^b^log_10_-transformed variable.

Because the CMgene cohort is a collection of several previous PET study projects, the scheduling of liver and thigh area imaging was different according to the study. However, neither timing of the liver nor thigh scan relative to the [^18^F]FDG injection time appeared as significant confounders for OGTT glucose AUC prediction when analyzed together with the Confirmatory model 1 and did not change interpretation of the results (data not shown). Moreover, the liver fat fraction could confound the measurement of insulin-stimulated liver GU because fat takes space in the liver cells [42]. Nevertheless, a subgroup analysis where liver GU was expressed per lean liver tissue yielded a model (*R*^2^ = 20%, *P* = .015) where liver GU was an independent predictor (Supplementary Table S4 [21]). In addition, visceral adipose tissue could modify the relationships observed in Table 2 through local effect on the lipid flux to the liver [13, 43]. However, visceral fat mass did not appear to be a significant predictor of OGTT glucose curves when included in a Confirmatory model 1, and both EGP (*P* < .001) and liver GU (*P* < .001) remained as significant predictors (Supplementary Table S5 [21]). The model performance improved slightly (*R*^2^ = 43%, *P* = 1.6E-8).

Last, even though there were no differences in fasting liver GU between insulin-sensitive and insulin-resistant individuals in our previous study [2], it is still possible that variation in basal hepatic GU could contribute to the OGTT glucose prediction. However, albeit limited by a small sample size, using the differences in liver GU, skeletal muscle GU, serum free fatty acids, and EGP between fasting and clamp from this previous study [2] for OGTT glucose prediction (*R*^2^ = 40%, *P* = .015) suggests that it is in fact the insulin action on hepatic GU (*P* = .053) and not variation in basal hepatic GU that contributes to the prediction of OGTT glycemia (Supplementary Table S6 [21]). In this substudy, the difference between clamp and fasting EGP had the highest standardized regression coefficient that is in line with the known important role of insulin-mediated suppression of EGP on glycemic control. In contrast, fasting measurements of EGP, hepatic or skeletal muscle GU, or antilipolytic insulin index did not predict OGTT glucose AUCs in this substudy (data not shown).

To understand better the contribution of different insulin secretion parameters on the OGTT glucose prediction, we performed an additional regression analysis including parameters from the mathematical modeling of OGTT data (Table 6). Inclusion of these parameters to the regression analysis, together with potential interaction effects from sex, resulted in a better-performing model (*R*^2^ = 52%, *P* = 6.3E-12), where EGP, glucose sensitivity, potentiation factor ratio, and antilipolytic insulin index were independent predictors for the OGTT glucose AUCs. Furthermore, as observed with Confirmatory model 1, the antilipolytic insulin index had an interaction with sex (lower coefficient in women).

**Table 6.** Regression model predicting oral glucose tolerance test glucose (OGTT) area under the curve from 0 to 120 min (AUC; mmol/L*min)*^a^*

	Unstandardized coefficients	95% CI		Standardized coefficients	*P* value
	B	Lower	Upper	β	
(Constant)	39.387	32.891	47.463		<.001
Endogenous glucose production (µmol/body weight kg/minute)	0.112	0.046	0.179	0.292	.003
Glucose sensitivity (pmol min^−1^ m^−2^ L mmol^−1^)*^b^*	−4.702	−8.065	−2.299	−0.351	.002
Rate sensitivity (pmol m^−2^ L mmol^−1^)*^a^*	−0.013	−0.085	0.063	−0.046	.726
Potentiation factor ratio*^b^*	−4.375	−8.318	−0.605	−0.279	.026
Hepatic GU (µmol L^−1^ tissue min^−1^)*^a^*	−0.438	−0.877	0.049	−0.149	.051
Skeletal muscle GU (µmol tissue kg^−1^ minute^−1^)*^a^*	−0.159	−0.561	0.143	−0.100	.355
Antilipolytic insulin index (pmol/L*mmol/L)*^b^*	3.134	0.683	5.317	0.296	.007
Insulin clearance clamp (L m^−2^ minutes^−1^)*^b^*	3.410	−5.222	12.364	0.104	.453
Insulin clearance OGTT (L m^−2^ minutes^−1^)*^b^*	−1.394	−6.862	4.324	−0.063	.629
Fasting triglycerides (mmol/L)*^b^*	3.706	0.278	6.566	0.231	.020
sex*glucose sensitivity (pmol min^−1^ m^−2^ L mmol^−1^)*^b^*	−2.923	−7.021	1.630	−0.941	.163
sex*rate sensitivity (pmol m^−2^ L mmol^−1^)*^a^*	0.007	−0.077	0.081	0.035	.860
sex*potentiation factor ratio*^b^*	4.525	−0.355	9.986	0.258	.083
sex*antilipolytic insulin index (pmol/L*mmol/L)*^b^*	−2.546	−5.299	0.718	−0.541	.078
sex*insulin clearance clamp (L m^−2^ minutes^−1^)*^b^*	−9.535	−20.898	1.234	−0.383	.089
sex*insulin clearance OGTT (L m^−2^ minutes^−1^)*^b^*	0.331	−6.709	9.398	0.010	.920
sex*fasting triglycerides (mmol/L)*^b^*	−5.625	−9.726	−1.330	−0.207	.009
Sex (M: 0; F: 1)	6.822	−3.547	15.886	1.077	.151

This analysis was performed to study if the inclusion of parameters from mathematical modeling of OGTT data would improve prediction of OGTT glucose AUC. Of the OGTT-derived parameters glucose sensitivity describes glucose-sensitive insulin secretion, rate sensitivity describes secretion response to rate of change in glucose concentration, and potentiation factor ratio describes insulin secretion response to potentiating factors, including incretins. The contribution of hepatic insulin clearance is higher in the OGTT insulin clearance measurement compared to the clamp insulin clearance due to hepatic first-pass insulin extraction. The standardized regression coefficients in the model serve as intuitive indices of effect size: higher absolute value for a coefficient indicates larger effect on glucose AUCs. OGTT modeling data was available for 111 participants.

Abbreviations: F, female; GU, glucose uptake; M, male; OGTT, oral glucose tolerance test.

^a^Square root transformed variable.

^b^log_10_-transformed variable.

In addition, we did separate regression analyses using the variables listed in Table 2 to predict OGTT glucose AUCs in each 30-minute interval: Proportions of variance explained for each interval were 17% for 0 to 30 minutes, 32% for 30 to 60 minutes, 40% for 60 to 90 minutes, and 37% for 90 to 120 minutes (*P* < .05 for all), reflecting the time course of insulin secretion and action [44]. Suppression of EGP and fasting triglycerides were significant predictors on all the intervals, whereas insulin secretion, hepatic GU, and the antilipolytic insulin index were significant predictors in the latter half of OGTT.

## Discussion

In this cross-sectional study, we showed by employing linear regression that insulin-stimulated hepatic GU was an independent predictor of OGTT glucose levels when accounting for several known factors contributing to glycemic control. Thus, our results suggest that measurement of hepatic GU during a HEC study with PET is a noninvasive measurement that yields physiologically meaningful information about insulin sensitivity of hepatic glucokinase activity. The ability to quantify hepatic GU directly using PET is a major strength over the traditional arteriovenous difference technique where studies of hepatic GU are based on measurements over the whole splanchnic region because catheterization of the portal vein is not feasible in humans. In addition, because the considerable increase in hepatic GU after an oral glucose delivery is based on the liver getting exposed to high glucose and insulin concentrations in the portal vein where they are initially released [14, 45], the robust association between hepatic GU and OGTT glucose_AUC_ in our study possibly indicates a significant role of first-pass hepatic glucose extraction in postprandial glycemic control.

Total rate of EGP can be considerably higher compared to hepatic GU during a HEC study as demonstrated by our study in pigs [8, 24] because both hyperinsulinemia and hyperglycemia are needed for net hepatic GU [46]. However, the phosphorylation of glucose entering the liver cells and the dephosphorylation of glucose-6-phosphate derived from gluconeogenesis, glycogen breakdown or cycling back from glycolysis are compartmentalized processes, which means that, for the measurement of hepatic GU, glucose phosphorylation dominates over dephosphorylation, allowing the use of irreversible [^18^F]FDG trapping for quantification [7, 8]. Compartmentalization of these processes is also demonstrated by the lack of correlation between the measured hepatic GU and EGP during insulin stimulation [22]. Nevertheless, incomplete suppression of glucose-6-phosphatase activity by insulin probably also contributes to the observed variation in hepatic GU [5, 8].

As expected, the participants with high glucose_AUC_ in our study showed skeletal muscle and adipose tissue insulin resistance compared to the group of low glucose_AUC_. Insulin secretion measured from OGTT was relatively similar in proportion to glucose levels in participants with high glucose_AUC_ suggesting that insulin resistance, and not insulin secretion capacity was the main driver of higher glucose_AUC_ in this group. In addition, hepatic GU was not different between the groups suggesting that insulin resistance of hepatic GU differs from skeletal muscle and adipose tissue as was shown in our previous study [22]. This difference may be related to the liver fat content or fatty acid flux from the visceral adipose tissue. The participants with high glucose_AUC_ had higher total insulin output and lower β-cell glucose sensitivity and potentiation factor ratio consistently with previous reports [35, 36].

Insulin clearance measured from the clamp study did not differ between participants with low and high glucose_AUC_, suggesting that peripheral insulin clearance was intact also among individuals with poorer glycemic control. In contrast, insulin clearance from OGTT, which reflects mostly hepatic insulin clearance, showed a decrease in participants with high glucose_AUC_. Insulin clearance has been shown to be associated with impaired glucose tolerance and obesity [47]. Insulin clearance during the clamp study was not an independent predictor of glucose_AUC_ in the primary regression model and in the analysis including parameters from the mathematical modeling of OGTT data. However, there was a statistical interaction between sex and clamp insulin clearance where women had a lower coefficient for the effect of insulin clearance on glucose_AUC_. Insulin clearance has been previously shown to be different between men and women [48, 49], likely because of differences in body composition [50], which might contribute to this observation.

In the current study, we found that fasting serum triglycerides predicted higher OGTT glucose_AUC_. This finding is in line with the results from a recent study by Tricò et al [17], where a low-grade lipid infusion during an OGTT impaired glucose tolerance, insulin sensitivity, and insulin clearance while increasing insulin secretion. These findings agree with a recent Mendelian randomization study by Zhu et al [39], which which suggested a bidirectional causal relationships between lipid traits and fasting insulin and HbA1c. It is possible that the serum triglyceride level provides further information regarding lipolytic activity and fatty acid flux affecting insulin sensitivity and glycemic control that is not quantified by the antilipolytic insulin index alone. This is because a considerable amount of circulating free fatty acids originate from hydrolysis of serum triglycerides [51] and much of the hepatic triglyceride synthesis relies on hepatic free fatty acid delivery independent of insulin [52].

This study has some limitations. First, we did not include patients with type 2 diabetes in this study because the available number of patients with these measures was too low for group comparisons and adjustment for the potential confounding from diabetes medication. Second, the study had considerably fewer women compared to men and the women tended to be older and were more insulin resistant, which are limitations of the current study. When we tested the effect of sex on the prediction of glucose_AUC_, we found that the antilipolytic insulin index, clamp insulin clearance, and triglycerides had or tended to have a lower coefficient for the prediction of glucose_AUC_ among women. The differential effect between sexes likely stems from differences in body composition. It has been recently shown that men have poorer antilipolytic effect of insulin compared to women in obesity [53], and insulin clearance is directly related to body muscle percentage but negatively to body fat percentage [50]. Moreover, a previous study by Mittendorfer et al [54] demonstrated that plasma very low-density lipoprotein (VLDL) triglyceride concentration was mainly determined by the VLDL triglyceride secretion rate in men but mostly by clearance rate in men and that both VLDL triglyceride secretion and clearance associated with body composition. Furthermore, menopause/women's age has been associated with reduced insulin clearance [49, 55] and lipolysis [56] and an increase in triglycerides [57], which may also contribute to our findings. According to some studies, oral contraceptives containing estradiol tend to induce insulin resistance, whereas progestins enhance insulin secretion [58-60]. On the other hand, menopausal hormone therapy improves insulin sensitivity and insulin clearance [61]. Neither the estradiol or progestin treatment appeared as significant predictors when added to the Confirmatory model 1 and had little effect on the model coefficients; however, we were not sufficiently powered to examine the potential effects in detail in the current study. Thus, the possible effects of the use of oral contraceptives, hormone therapy, and menopause on glycemic control need to be more carefully examined in future studies.

In addition, the majority of the variation in OGTT glucose AUCs was unexplained by our model. The lengthy interval between the OGTT and PET study because of logistical reasons may have added noise that reduced the amount of variation explained by the models to some extent. However, based on the standardized coefficients of the primary model, whereas insulin secretion, EGP, and fasting triglycerides were the most important determinants of OGTT glucose curves, hepatic GU had approximately similar effect size as skeletal muscle GU. The high standardized coefficient for EGP in the primary model agrees to the known role of EGP as a key determinant of glucose tolerance [62]. Further, even though skeletal muscle GU was not a statistically significant predictor for OGTT glucose_AUC_ in our study this is probably more related to the differences in body composition and muscle mass than an actual lack of effect. This is because M-value or R_d_, which mostly reflect skeletal muscle GU but also measure GU into other tissues, were significant predictors when used in the analysis instead of muscle GU and the difference in the standardized coefficient for skeletal muscle and the other key predictors was small.

A major proportion of the unexplained variation in OGTT AUCs in our study are likely related to the glucose route of delivery. These factors include gastric emptying and glucose absorption rate, where variation has been shown to affect OGTT glucose levels [63] as well as the effect of portal glycemia after glucose ingestion on hepatic GU and suppression of EGP [45]. The role of incretins on the OGTT glucose curves was also not directly assessed. These factors need to be studied in more detail in future studies employing [^18^F]FDG-PET in which the tracer is given via oral route, an approach that has been recently validated in mice [64].

In conclusion, these results suggest that the measurement of hepatic GU during euglycemic hyperinsulinemia gives useful insight about insulin's ability to promote hepatic GU in physiologic context. In addition, the robust independent association of hepatic GU with OGTT glycemia in the regression analysis suggests that first-pass hepatic glucose extraction is physiologically relevant for postprandial glycemic control. This supports the use of this measurement when evaluating current and potential treatments of conditions involving dysregulated hepatic glucose metabolism and in the better understanding of the pathophysiology leading to reduced glucose tolerance. Last, the role of serum triglycerides in glycemic control needs to be studied further from both mechanistic and clinical perspective because the finding that triglycerides associate with increased OGTT glucose AUCs suggests that serum triglycerides may contribute to the progression of prediabetes and type 2 diabetes.

## Data Availability

Restrictions apply to the availability of some or all data generated or analyzed during this study to preserve patient confidentiality or because they were used under license. The corresponding author will on request detail the restrictions and any conditions under which access to some data may be provided.

## Clinical Trial Information

ClinicalTrials.gov Identifier: NCT03310502.

## Abbreviations

AUC: area under the curve
BMI: body mass index
EGP: endogenous glucose production
[^18^F]FDG: 2-deoxy-2-[^18^F]fluoro-D-glucose
GU: glucose uptake
HbA1c: hemoglobin A1c
HEC: hyperinsulinemic euglycemic clamp
HOMA-IR: homeostatic model assessment for insulin resistance
OGTT: oral glucose tolerance test
PET: positron emission tomography
VLDL: very low-density lipoprotein
